# Suppression of class I compensated cell enlargement by *xs2* mutation is mediated by salicylic acid signaling

**DOI:** 10.1371/journal.pgen.1008873

**Published:** 2020-06-25

**Authors:** Ushio Fujikura, Kazune Ezaki, Gorou Horiguchi, Mitsunori Seo, Yuri Kanno, Yuji Kamiya, Michael Lenhard, Hirokazu Tsukaya

**Affiliations:** 1 Department of Biological Sciences, Graduate School of Science, The University of Tokyo, Japan; 2 Graduate School of Science, The University of Tokyo, Japan; 3 Department of Life Science, College of Science, Rikkyo University, Japan; 4 RIKEN Center for Sustainable Resource Science, Japan; 5 Institut für Biochemie und Biologie, Universität Potsdam, Potsdam-Golm, Germany; 6 Okazaki Institute for Integrative Bioscience, Japan; The University of North Carolina at Chapel Hill, UNITED STATES

## Abstract

The regulation of leaf size has been studied for decades. Enhancement of post-mitotic cell expansion triggered by impaired cell proliferation in Arabidopsis is an important process for leaf size regulation, and is known as compensation. This suggests a key interaction between cell proliferation and cell expansion during leaf development. Several studies have highlighted the impact of this integration mechanism on leaf size determination; however, the molecular basis of compensation remains largely unknown. Previously, we identified *extra-small sisters* (*xs*) mutants which can suppress compensated cell enlargement (CCE) via a specific defect in cell expansion within the compensation-exhibiting mutant, *angustifolia3* (*an3*). Here we revealed that one of the *xs* mutants, namely *xs2*, can suppress CCE not only in *an3* but also in other compensation-exhibiting mutants *erecta* (*er*) and *fugu2*. Molecular cloning of *XS2* identified a deleterious mutation in *CATION CALCIUM EXCHANGER 4* (*CCX4*). Phytohormone measurement and expression analysis revealed that *xs2* shows hyper activation of the salicylic acid (SA) response pathway, where activation of SA response can suppress CCE in compensation mutants. All together, these results highlight the regulatory connection which coordinates compensation and SA response.

## Author summary

Leaves are determinate organ and size of leaves are determined by intrinsic and extrinsic cues. Cell proliferation and post-mitotic cell expansion should be coordinated during leaf morphogenesis to develop appropriate size depending on its developmental programs. Recent studies highlighted the existence of integrated mechanism which coordinates cell proliferation and cell expansion during leaf development. Compensation, which is enhanced post-mitotic cell expansion accompanied by a significant decrease in cell number during leaf organogenesis, is one of the clues for such coordination. However, the molecular mechanisms linking cell proliferation and cell expansion are still poorly understood. Previously, we reported *extra-small sisters 2* (*xs2*) mutation caused specific defect in cell expansion and it suppressed increased post-mitotic cell enlargement in *angustifolia3 (an3)* mutant, which exhibits typical compensation. Here we identify the affected gene of *xs2* mutant encodes a member of cation calcium exchanger which is believed to be involved in cation homeostasis within cells. Loss of function of this protein causes hyper accumulation of salicylic acid (SA) and increased expression of pathogen related genes. Physiological and genetic studies revealed activated SA signal transduction reduced cell size. It suppressed post-mitotic cell expansion in several compensation mutants not only *an3* but partially suppressed in another type of compensation mutant which increases size of mitotic cells. This finding suggests post-mitotic cell expansion pathway is regulated in common by SA-dependent signaling and by compensation signaling during leaf development.

## Introduction

Understanding how organ size is regulated in plants has remained as a fundamental question in the field of plant science over the last few decades. The plant leaf is one of the most suitable model systems for studying organ size determination, since leaves show constant size and shape under a given growth condition. Many studies have demonstrated complex regulatory networks for organ size determination in *Arabidopsis thaliana* (L.) Heynh. (Arabidopsis, hereafter). As leaves are a determinate organ, which is produced by limited cell proliferation, the final leaf size is determined by the total number and average size of cells within leaves. Vigorous cell proliferation occurs at the base of the young leaf primordia, then proliferative zone of primordia is spatially differentiated at the junction region between the leaf blade and leaf petiole. Then, cells that become displaced distally away from the base gradually lose their proliferating activity along the proximal-distal axis [1, 2]. Cells that exit from this leaf meristem region start post-mitotic cell expansion, which is accompanied by massive vacuolation [1–9]. Several studies have highlighted the phenomenon of “compensation”, which refers to a decrease in cell number accompanied with a significant increase in cell size, caused by a mutation or ectopic expression of a particular transgene. These findings suggest that the two spatially separated events, cell proliferation and cell expansion are highly coordinated during leaf development [7, 10–19]. Kinematic analysis of several compensation-exhibiting mutants revealed that abnormal cell enlargement, termed “compensated cell enlargement (CCE)” can be classified into three classes based on their way of development [13, 20]. For instance, CCE in *angustifolia3* (*an3*), *fugu2*/*fasciata1* (*fugu2/fas1*) and *erecta* (*er*) occurs by enhanced post-mitotic cell expansion activity (class I), while an extended post-mitotic cell expansion period occurs in *fugu5* (class II). An increased size of dividing cells contributes to a larger cell phenotype in a *KIP-RELATED PROTEIN 2* (*KRP2*) overexpressor (*KRP2ox*) (class III). Another difference is that compensation is mediated in a cell-autonomous and a non-cell-autonomous manner in the *KRP2ox* and *an3*, respectively, as Kawade *et al*. have demonstrated [21].

A detailed developmental context of compensation, especially how cell number is reduced, has been characterized in recent studies. For example, in the class I compensation mutant *fugu2/fas1* an *ATAXIA TELANGIECTASIA MUTATED* (*ATM*)-dependent DNA damage response contributes to cell cycle delay [22]. On the other hand, Ferjani *et al*. [23, 24] showed that a class II compensation mutant *fugu5* exhibits hyper-accumulation of cytosolic pyrophosphate and decreased levels of sucrose due to loss of AVP1 (vacuolar H^+^-pyrophosphatase) activity, leading to impaired cell proliferation. Although previous study suggests that a key trigger of compensation induction is a significant reduction in cell number below a certain threshold level [25], little is known about the regulatory mechanisms underlying CCE other than class III compensation.

Regarding class III compensation, the loss of function mutant of *DE-ETIOLATED 3* (*DET3*) gene encoding the V-ATPase suppressed CCE of *KRP2ox* without any effects on cell proliferation, suggesting that CCE in *KRP2ox* plants requires V-ATPase activity [18, 19]. Interestingly, introduction of the *det3* mutation into another compensation mutant, *fugu2* (class I) or *fugu5* (class II) did not suppress CCE, indicating that the cell expansion pathways that are activated by compensation in the three classes are distinct. To study the mechanism of CCE further, we identified mutants, so called *extra-small sisters* (*xs*), that have a specific defect in cell expansion [26]. Double mutant analysis combining *xs* mutants and *an3*, a class I compensation mutant, was carried out to evaluate the genetic interaction between *xs* mutants and *an3*. Interestingly, some of the *xs* mutations completely suppressed CCE in the *an3* background while cell number was not affected. This suggests that CCE in class I compensation occurs by massive activation of a cell expansion pathway that is required for normal cell expansion during leaf development. To understand the regulatory mechanisms of CCE, further characterization of *xs* mutants including molecular cloning of *XS* genes has been required. Therefore, in this study we characterized the *xs2* mutant that shows strong inhibition in cell expansion. Our results revealed that *XS2* encodes a *CATION CALCIUM EXCHANGER 4* (*CCX4*) and *xs2* mutant accumulates increased levels of the phytohormone salicylic acid (SA), leading to hyper-activation of the SA response and to impaired cell expansion.

SA is known to be a key signal molecule in activating defenses, acquiring resistance to pathogens, and cell death during plant-pathogen interactions in several species [27, 28]. NPR1 (NONEXPRESSOR OF PR GENES 1) activates the SA-controlled systemic acquired resistance (SAR) pathway. In addition to defense responses, several studies highlight complex roles of SA in cell fate control, such as regulation of organ growth, cell division, cell enlargement, DNA endoreduplication and cell death [29–32]. In this study, we found that an *xs2 npr1* double mutant shows a normal rosette phenotype in terms of cell size and number, suggesting that SA-dependent inhibition of cell expansion in *xs2* is mediated by NPR1 signal transduction. These results provide novel insights into compensation and SA signaling during leaf development.

## Results

### Loss of *XS2* function can suppress CCE in compensation-exhibiting mutants *an3*, *er* and *fugu2* but not in *KRP2ox*

**Fig 1:**
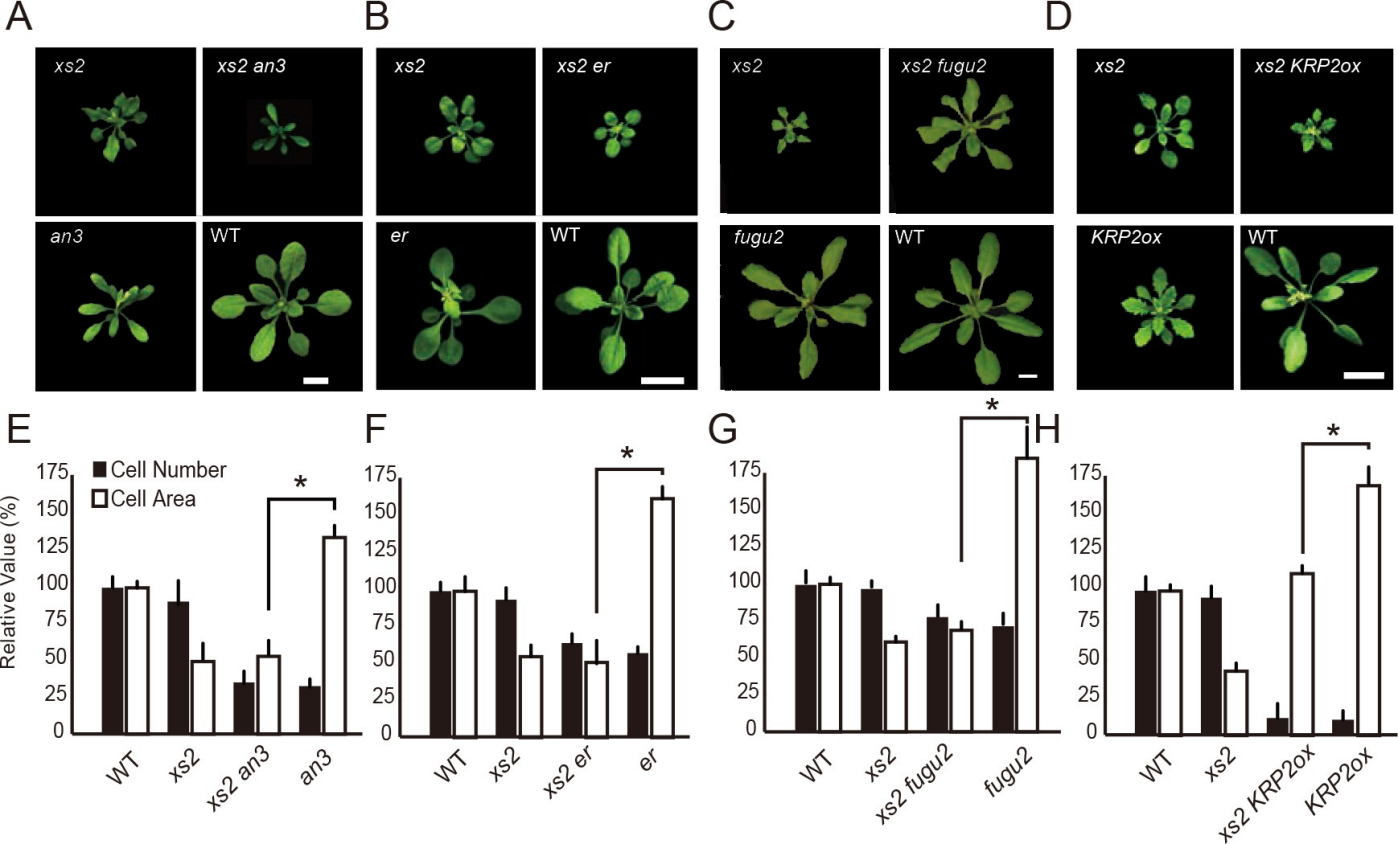
Genetic interaction between *xs2* and compensation-exhibiting mutants. (A to D) Rosette phenotype of WT, *an3-3*, *xs2* and compensation-exhibiting mutants *er*, *fugu2-1* and *KRP2ox*. Plants were grown for three weeks under a 16-h-light/8-h-dark fluorescent illumination cycle at 22ºC. Scale bars are 10 mm. (E to H) Cell size and cell number from first layer of palisade cells. First leaves from three-weeks-old plants were used for observation. (n ≥ 240 cells from more than eight leaves). Means + SD. *, significantly different at p < 0.05 (with Bonferroni correction for comparisons to WT).

We previously reported that the *xs2* mutation can suppress CCE in the compensation-exhibiting mutant *an3* (Fig 1A–1E, [26]). There are several types of mutants that exhibit compensation with a different developmental basis [13, 21]. This raises the question whether the *xs2* mutation can suppress CCE only in *an3* or can also suppress it in other compensation-exhibiting mutants. To address this, double mutants between *xs2* and *er*, *fugu2* and *KRP2ox* plant, which all show a typical compensation phenotype (Fig 1, [13, 22]), were constructed. *er* leaves showed a significant decrease in cell number and an increase in cell size compared to wild-type (WT) leaves. While the *xs2 er* double mutant leaves had fewer cells as seen in parental *er* mutants, they had smaller cells than *er* single mutants and like those in *xs2*, indicating that the *xs2* mutation can also suppress CCE in the *er* mutant without affecting cell number (Fig 1B–1F). Similarly, CCE in the *xs2 fugu2* double mutant was also suppressed, while the number of cells was not affected (Fig 1C–1G). These results suggested that CCE in *an3*, *er* and *fugu2* might occur through the same regulatory pathway of cell expansion mediated by *XS2*. By contrast, size of cells in the *xs2 KRP2ox* double mutant showed an intermediate phenotype between the parents, suggesting that the CCE in *KRP2ox* occurs through an *XS2*-independent regulatory pathway (Fig 1D–1H).

### *xs2* has a deletion in *CCX4*

**Fig 2:**
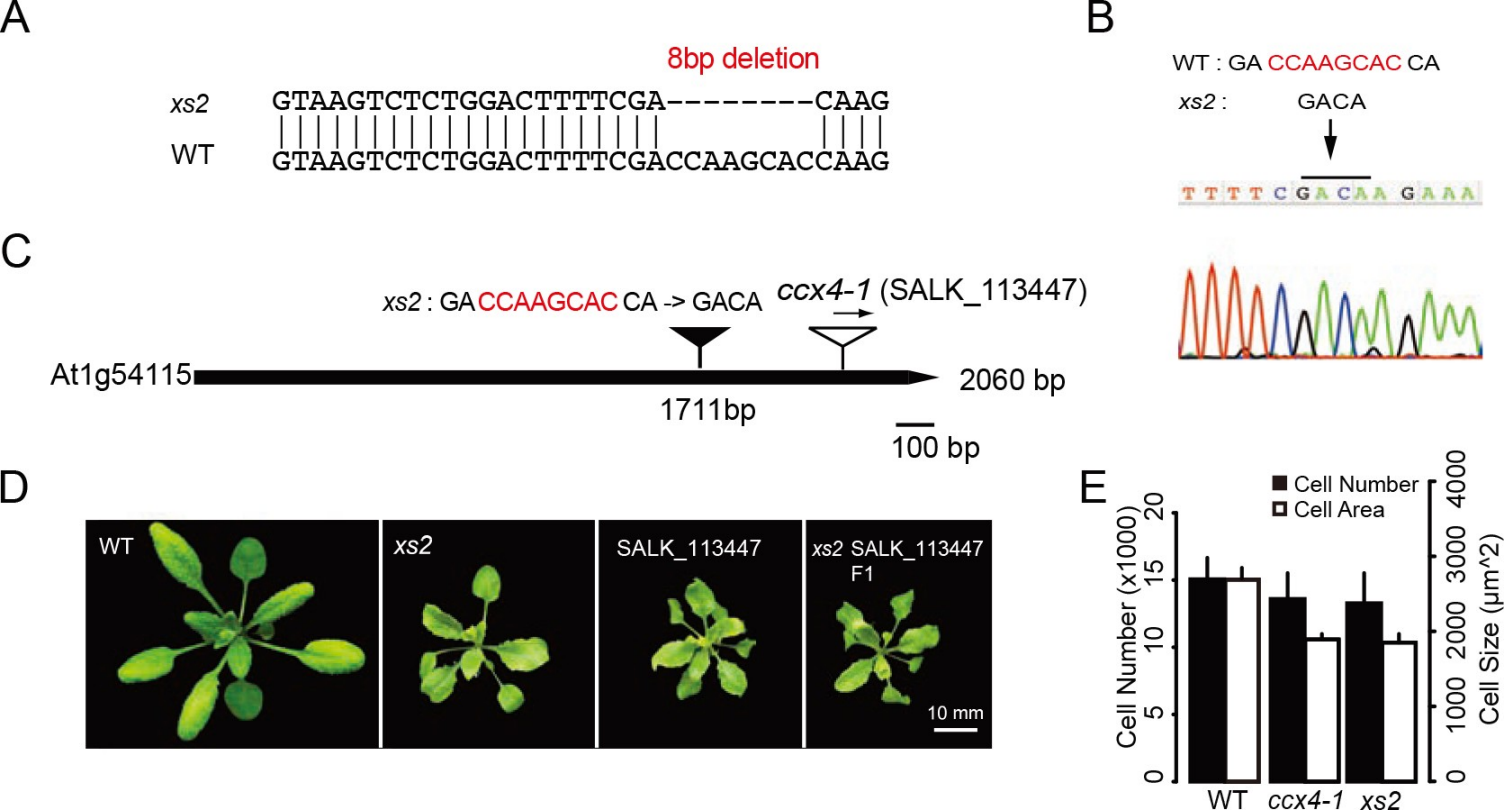
*XS2* corresponds to At1g54115. (A) An 8-bp deletion was found within At1g54115 in *xs2*. (B) Sequence spectra of sense strand of At1g54115 region in *xs2* mutant. (C) Schematic representation of the At1g54115 locus and the locations of the deletion in the *xs2* mutant (black triangle) and T-DNA insertion (white triangle) in *ccx4-1* mutant. Deletion found in the *xs2* mutant is represented with red colored characters. (D) Complementation test between *xs2* and *ccx4-1*. (E) Cellular phenotype in *xs2*, *ccx4-1* and WT. Means + SD. First leaves from three-week-old plants were used for observation. (n ≥ 240 cells from eight leaves). Scale bar is 10 mm.

To determine the molecular identity of the *XS2* gene, a map-based cloning approach was employed. Fine mapping and sequence analyses revealed an 8-bp deletion in the exon of At1g54115 in the *xs2* mutant (Fig 2A and 2B and S1 Fig), which encodes a CATION CALCIUM EXCHANGER 4 (CCX4), a putative endomembrane H^+^-dependent K^+^ transporter (Fig 2C). It is suggested that AtCCX4 has an activity of Na^+^, K^+^ and Mn^2+^ transport when expressed in yeast [33]. This loss-of-function mutation occurs in the region that is predicted as sodium/calcium exchanger membrane domain and causes a frame shift that might lead to dysfunction of CCX4. Next, to determine if loss of CCX4 function causes the *xs2* phenotype, *ccx4-1*, a T-DNA insertion mutant allele was characterized (Fig 2C–2E, [33]). *ccx4-1* had a smaller rosette phenotype due to an impaired cell expansion and showed no significant difference in cell number compared to WT as seen in the *xs2* mutant (Fig 2D). To confirm that *CCX4* is the responsible gene of *xs2*, *xs2* was crossed with *ccx4-1* and the phenotypes of the F_1_ progeny were characterized. The F_1_ plants showed a similar phenotype to the parental *xs2* or *ccx4-1* (Fig 2D, S1C Fig). Double mutants between *an3* and *ccx4-1* showed an impaired cell expansion phenotype like that in the *xs2 an3* double mutant (S3B and S3C Fig). Taken together, we conclude that the responsible gene of *xs2* is *CCX4*. RT-PCR analysis showed that the accumulation of transcript including the 3' untranslated region was decreased in *ccx4-1*, while the *xs2* mutant showed comparable expression levels as WT (S1A and S1B Fig), suggesting that nonsense-mediated mRNA decay does not occur in the *xs2* mutant.

### *xs2* and *ccx4-1* show constitutively activated salicylic acid signaling

We confirmed that loss of CCX4 function causes specific defects in cell expansion, raising the question how it does so. Considering that *xs2* shows several defects not only in cell expansion but also in plant development such as decreased endoreduplication levels [26], we noticed a similarity in such developmental defects between *xs2* and mutants of genes involved in pathogen response. For example, a constitutive SA-response mutant of Ca^2+^/calmodulin (CaM)-regulated transcription factor shows hyper accumulation of transcripts of pathogenesis-related (PR) genes and also shows a smaller rosette phenotype as *xs2* [34]. It is also known that SA responses including NPR1-mediated pathogen/defense signaling affect cell expansion and that lower accumulation of SA causes an increased ploidy level [30, 32, 35].

**Fig 3:**
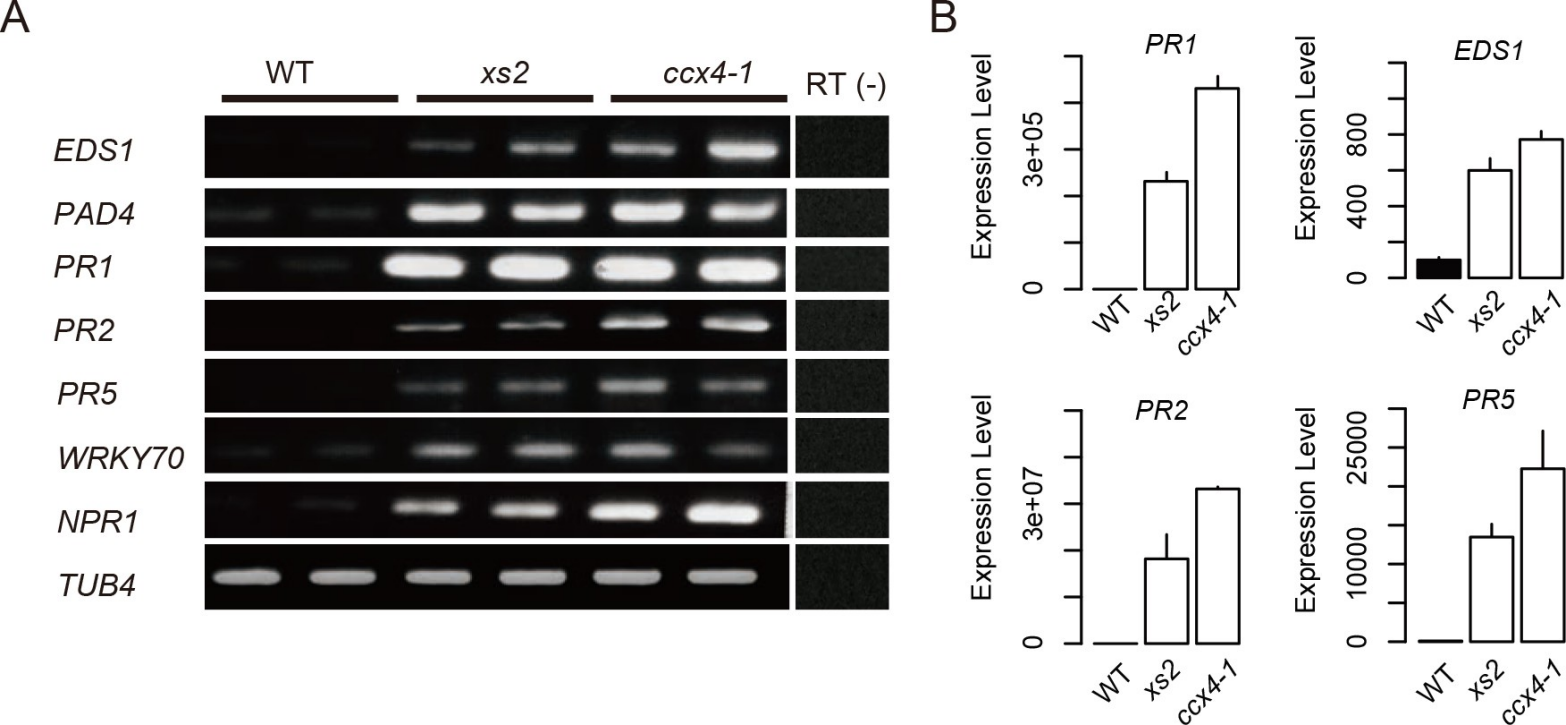
Expression of pathogen defense response related genes in WT, *ccx4* and *xs2* mutants. (A) RT-PCR and (B) qRT-PCR analyses are shown for salicylic acid signaling related genes in WT, *ccx4-1* and *xs2* mutants. Total RNA was prepared from the first set of leaves from ten-day-old plants and used for expression analysis. The *TUB4* gene was used for an internal control. All values were normalized against the expression level of the *TUB4* gene. Data is from three biological replicates for qRT-PCR analysis. Error bars indicate + SD.

Thus, we hypothesized that pleiotropic phenotypes observed in *xs2* might be due to hyper-activation of SA signaling. First, we determined the expression levels of genes involved in SA biosynthesis or pathogen/defense response in *xs2*, *ccx4-1* and WT. Semi-quantitative RT-PCR analysis revealed that expression of the three SA-dependent SAR markers *PR1*, *PR2* and *PR5* is dramatically increased in *xs2* and *ccx4-1* compared to WT (Fig 3A). Expression of *ENHANCED DISEASE SUSCEPTIBILITY 1* (*EDS1*) and *PHYTOALEXIN DEFICIENT 4* (*PAD4*), key regulators for basal and effector-triggered Toll-interleukin1-receptor domain NLR immunity [36], was increased in *xs2* and *ccx4-1* mutants compared to WT. The same is true for the expression of *NPR1*, a positive regulator of the SA-dependent signaling and SAR, and the WRKY transcription factor gene *WRKY70*, which functions as a convergence node of integrating signals from SA and jasmonic acid (JA)-dependent defense pathways (Fig 3A) [37, 38]. Our qRT-PCR confirmed massive overaccumulation of *EDS1*, *PR1*, *PR2* and *PR5* in *xs2* and *ccx4-1* mutants (Fig 3B). These results suggest that the SA response is constitutively activated in *xs2* and *ccx4-1* mutants.

### *xs2* and *ccx4-1* accumulate high levels of reactive oxygen species

Many studies have shown that SA induces the accumulation of reactive oxygen species (ROS) in plants and causes oxidative damage including programmed cell death (PCD) [39–42]. Considering that *xs2* and *ccx4-1* exhibit SA response in terms of gene expression, we carried out trypan blue staining to assess PCD levels in the first set of leaves of WT, *xs2* and *ccx4-1*. *xs2* and *ccx4-1* mutants showed strong staining within leaves (S3A Fig). This suggests that *xs2* and *ccx4-1* exhibit massive ROS production and induce a hypersensitive response (HR). Interestingly, *xs2* and *ccx4-1* showed no obvious defects in leaf senescence, an alternative form of PCD (S3C Fig).

### *xs2* and *ccx4-1* are SA hyper accumulation mutants

Expression analysis and trypan blue staining suggested that *xs2* and *ccx4-1* mutants have a constitutively activated SA-response. To get more insight into phytohormone levels, we determined endogenous levels of SA, auxin (indole-3-acetic acid, IAA), abscisic acid (ABA), gibberellin (GA_1_ and GA_4_), JA, jasmonic acid-isoleucine (JA-Ile), cytokinin, and cytokinin derivatives (isopentenyladenine [iP], dihydrozeatin [DHZ], *trans*-zeatin [tZ]) in WT, *xs2*, *ccx4-1*, and *an3* plants by using liquid chromatography-electrospray ionization tandem mass spectrometry (LC-ESI-MS/MS) (Table 1). Endogenous levels of SA were significantly higher in *xs2* and *ccx4-1* than in WT, while *an3* showed a similar value to WT. In contrast, accumulation of JA and JA-Ile was dramatically reduced in *xs2* and *ccx4-1* mutants compared to WT. These results confirm that *xs2* and *ccx4-1* highly accumulate SA.

**Table 1:** Comprehensive Analysis of Phytohormones in Wild-type, *xs2*, *ccx4-1*, and *an3* Mutant Plants.

Hormones (ng/gDW)	WT	*xs2*	*ccx4-1*	*an3*
SA	2059.4 ± 161.3	4038.8 ± 139.8 *	5333.7 ± 1313.9 *	2470.5 ± 189.7 *
IAA	99.4 ± 4.3	68.2 ± 2.7 *	68.5 ± 1.6 *	87.3 ± 11.0
ABA	52.9 ± 19.4	35.7 ± 0.1	39.1 ± 1.4	41.2 ± 2.2
GA1	nd	nd	nd	nd
GA4	3.8 ± 0.4	2.1 ± 0.1 *	2.1 ± 0.1*	4.3 ± 0.5
JA	57.2 ± 18.1	10.0 ± 5.9 *	9.2 ± 2.9 *	76.5 ± 20.8
JA-lle	9.3 ± 4.7	1.1 ± 0.7 *	0.6 ± 0.1 *	5.5 ± 1.5
iP	0.6 ± 0.1	1.2 ± 0.1	1.2 ± 0.2	0.6 ± 0.1
DHZ	nd	nd	nd	nd
tZ	nd	nd	nd	nd

Data represent the mean ± SD of three experiments. (nd, not detected; DW, dry weight

*P < 0.05)

### SA response suppresses cell expansion and CCE in *an3*, *er* and *fugu2* mutants, but not in *KRP2ox*

To gain more insight into the relationship between SA and cell expansion, we characterized the phenotype of *defense no death 1* (*dnd1*) mutant, since it exhibits similar phenotypic defects to *xs2*. The *dnd1* mutant shows constitutive systemic resistance and elevated levels of SA [43]. *DND1* encodes a CYCLIC NUCLEOTIDE-GATED ION CHANNEL2 (AtCNGC2), which is involved in passage of Ca^2+^, K^+^ and other cations across the plasma membrane [44]. The *dnd1* mutant is defective in HR cell death, but retains characteristic responses such as enhanced resistance against a broad spectrum of virulent fungal, bacterial and viral pathogens, including activated induction of pathogenesis-related gene expression [45]. Although it is suggested that the SA response limits cell expansion, the detailed cellular phenotype of this mutant has been unclear. If the increased level of SA is a critical cue for suppression of cell expansion, cell expansion in this mutant should be impaired. As expected, *dnd1* mutant had significantly smaller cells than WT, similar to *xs2* mutants (S4 Fig). This supports the idea that hyper accumulation of SA or activated SA response suppresses cell expansion.

**Fig 4:**
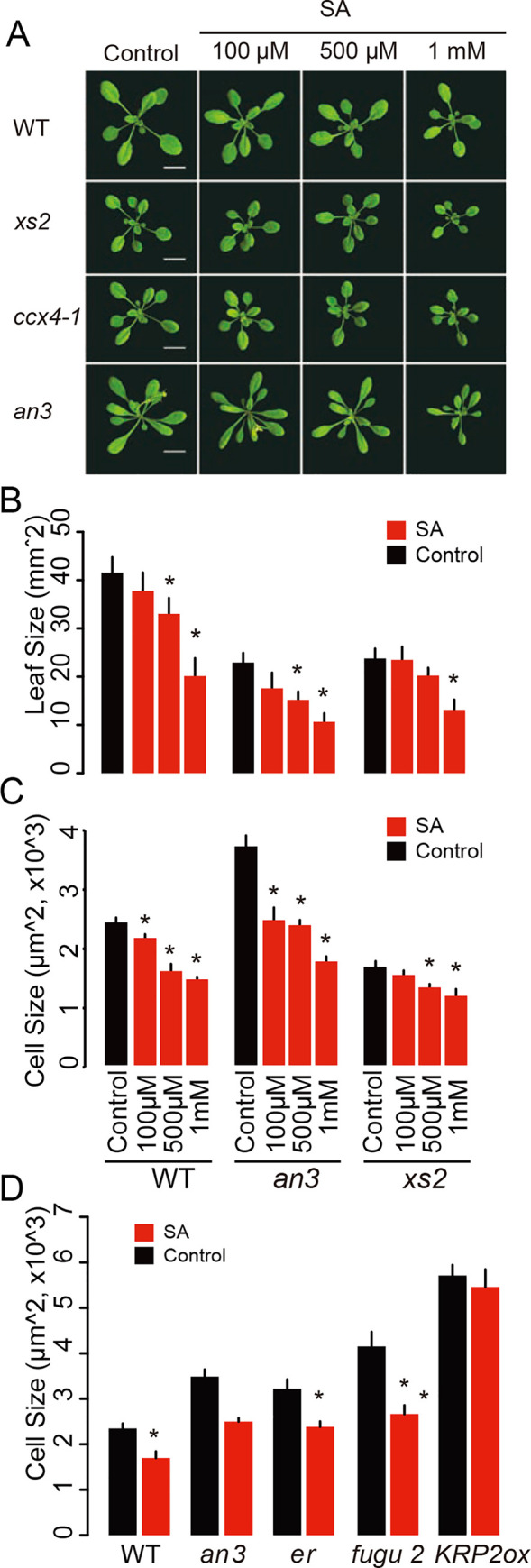
Effect of salicylic acid treatment on leaf development in WT, *xs2*, *ccx4* and *an3* mutants. (A) Rosette phenotype of plants treated with SA. Plants were grown for three weeks under a 16-h-light/8-h-dark fluorescent illumination cycle at 22ºC. Bars: 10 mm. (B) Leaf size and (C) leaf cell size in WT, *xs2* and *an3* mutants treated with salicylic acid with indicated concentration. Black bar plots represent control. (D) Cell size of SA treated WT, *an3*, *er*, *fugu2* and *KRP2ox*. SA solution at 1 mM was supplied every day. (B to D) Plants were grown for three weeks under a 16-h-light/8-h dark fluorescent illumination cycle at 22ºC. First leaves from three-week-old plants were used for observation. (n ≥ 240 cells from more than eight leaves). Means + SD. *, significantly different at p < 0.05 (with Bonferroni correction).

As shown above, the *xs2* mutation can suppress CCE in *an3* and other compensation-exhibiting mutants. If this suppression of CCE by *xs2* results from the hyper-activation of SA response, exogenous supply of SA is expected to result in a similar suppression of CCE as the *xs2* mutation. To address this possibility, *xs2*, *an3* and WT were treated with exogenous SA (Fig 4). Leaf size was significantly decreased by SA in a dose-dependent manner via a suppression of cell expansion (Fig 4A–4C). The inhibitory effect of SA on cell expansion was strongest in *an3* (52.7% reduction), while *xs2* showed a mild decrease in cell size (29.7%). Interestingly, sizes of cells in *an3* and WT reached almost the same value after treatment with 1 mM SA, while control *an3* had about 50% larger cells than WT (Fig 4C). This supports the idea that impaired cell expansion observed in *xs2* results from hyper-accumulation of SA and this SA-mediated suppression of cell expansion inhibits CCE in *an3*. As shown in Fig 1, CCE was suppressed not only in *an3* but also in *er* and *fugu2* by introducing *xs2* mutation. Thus, a next question is whether SA treatment can suppress CCE also in other compensation exhibiting mutants or not. To address this, we evaluated the effect of SA on cell expansion in *er*, *fugu2* and *KRP2ox* plants. Exogenous supply of 1 mM SA led to smaller cells in *er* and *fugu2* (Fig 4D). This result indicated that the cell expansion pathway(s) whose activation underlies compensation in *an3*, *er* and *fugu2* are also suppressed by SA. Remarkably, *KRP2ox* plants were not affected by SA in terms of cell size. This result is consistent with the fact that the *xs2* mutation could not suppress CCE in *KRP2ox*. Taken together, CCE in *KRP2ox* is activated by a SA-independent regulatory network of cell expansion.

### Inhibition of CCE in *xs2* was mediated by SA-dependent NPR1 signaling

**Fig 5:**
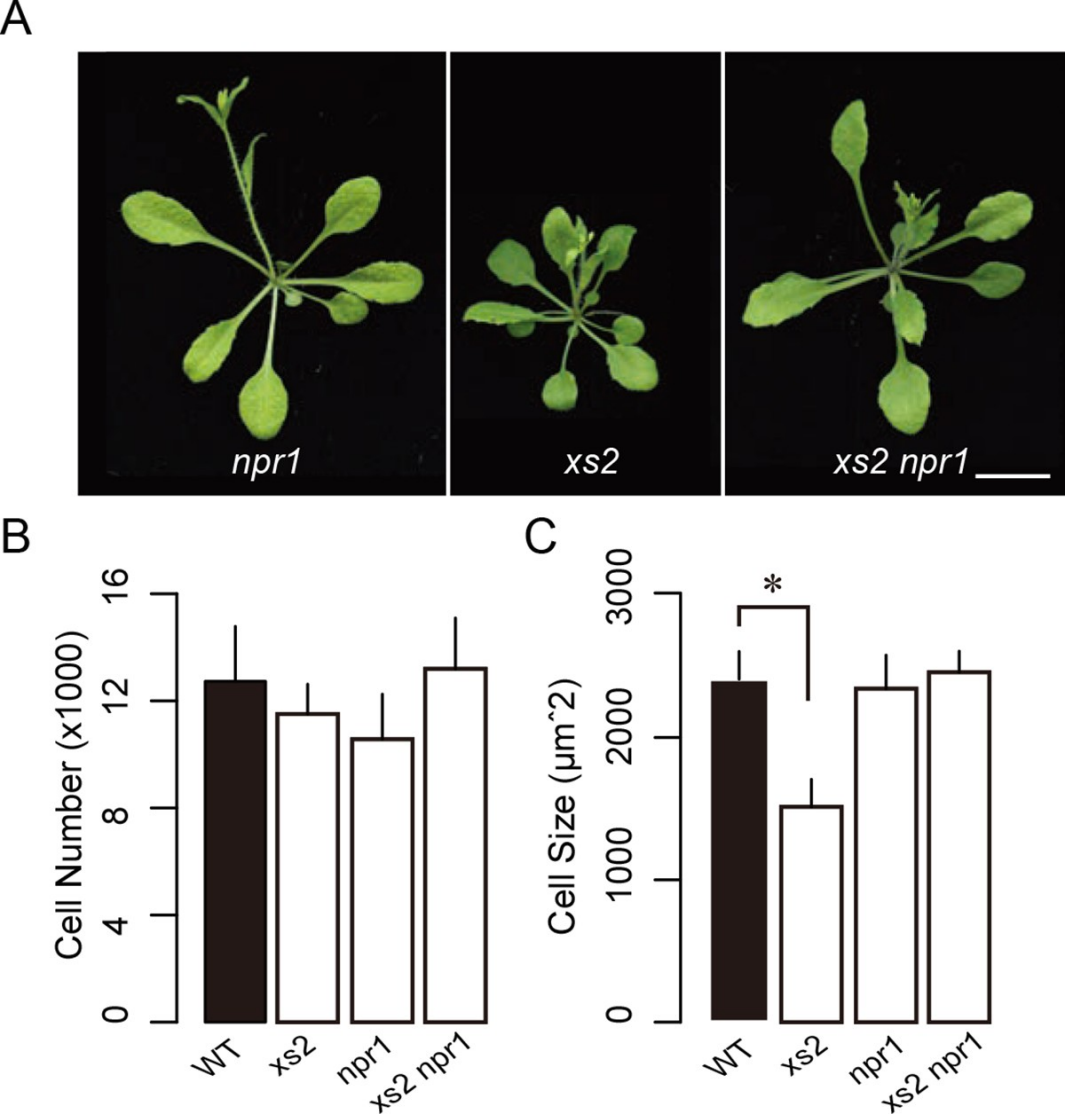
Characterization of the *xs2 npr1* double mutant. (A) Rosette phenotype of *xs2*, *npr1* and *xs2 npr1* mutants. Plants were grown for three weeks under a 16-h-light/8-h-dark fluorescent illumination cycle at 22ºC. Bars: 10 mm. (B) Estimated cell number and (C) cell size in WT and *xs2*, *npr1* and *xs2 npr1* mutants. First leaves from three-week-old plants were used for observation. (n ≥ 240 cells from more than eight leaves). Means + SD.

As shown above, activated SA response suppresses cell expansion in *xs2*. To determine how it does so, we assessed the role of the key SA-response factor *NPR1* in repressing cell expansion in *xs2*. To address this, we produced an *xs2 npr1* double mutant and evaluated the effect of the *npr1* mutation on cell expansion, since mutation in the *NPR1* gene blocks the induction of SAR by SA (Fig 5). The *npr1* mutant showed a slight decrease in cell number in comparison with WT, while cell size in *npr1* was unchanged (Fig 5B and 5C). Interestingly, a significant defect in cell size observed in the *xs2* mutant was restored in the *xs2 npr1* double mutant with a normal number of cells. This indicates that suppression of cell expansion in *xs2* was mediated via NPR1-dependent signal transduction downstream of SA.

## Discussion

In the last four decades, many reports have revealed mechanisms regarding plant organ size regulation [8, 10, 12, 14]. The phenomenon of compensation has been a key clue to understanding the integrated regulatory network between cell proliferation and cell expansion during organ development. To date, several studies have shown that defects in cell proliferation trigger compensation; however, little is known about which cell expansion pathway is activated in CCE. The phytohormone auxin was suggested to be involved in CCE in *fugu5*, a class II compensation mutant [46]. It is also reported that CCE in the class-III compensation-exhibiting mutant *KRP2ox* is mediated by V-ATPase activity [18, 19]. However, the mechanisms of CCE in class I compensation has been unclear. In this study, we showed that loss of CCX4/XS2 function suppresses CCE in class I compensation by causing an increased accumulation of SA and activated SA signaling. This indicates that activated SA signaling and class I compensation regulate common cell expansion pathways in opposite directions.

To understand the CCE suppression property of the *xs2* mutant, we characterized the *XS2* gene. The *xs2* mutant allele carries an 8-bp deletion that causes a frame shift within *CCX4*, encoding a member of subfamily of cation transporters [47, 48]. Our semi-quantitative RT-PCR analysis and subsequent qRT-PCR analysis further revealed that the expression of genes involved in SA-dependent defense response was up-regulated in developing leaves in *xs2* mutants (Fig 3). High levels of *EDS1* and *PAD4* expression, known as upstream activators of pathogen-induced SA accumulation, also supported that *xs2* has an activated SA response. Furthermore, our biochemical approach revealed that both the *xs2* and *ccx4-1* mutants accumulated high levels of SA (Table 1).

Several studies suggested that SA signaling affects cell enlargement and endoreduplication via recruiting multiple signaling pathways in developing leaves [30–32, 49–51]. Our genetic and biochemical approach demonstrated that the impaired cell expansion phenotype observed in *xs2* mutant results from activated SA signal transduction mediated by NPR1 (Table 1, Fig 5). It is known that increased ROS levels induce SA biosynthesis, and ROS production and SA biosynthesis form a positive amplification loop. NPR1 takes part in sensing the intracellular redox state that is modulated by SA-ROS loop [50, 52–55].

*CCX3* is known to be involved in K^+^, Na^+^ and Mn^2+^ transport within plant cells and *CCX4* might have similar functions to *CCX3*, since *CCX3* and *CCX4* arose from gene duplication [33]. It is also suggested that *CCX3* is involved in ROS signaling. Indeed, ectopic expression of Arabidopsis *CCX3* in tobacco plants showed higher protein oxidation state than controls [33]. This might result from disrupted metal concentrations derived by ectopic *CCX3* expression within cells, since regulation of metal concentration is critical for plant antioxidant regulations [33, 56]. Thus, it can be interpreted that loss of *CCX4* function causes altered redox state within cells and this causes activated ROS signaling and SA biosynthesis via NPR1. The loss of CCX3 and CCX4 functions has previously been reported to cause no visible phenotype in Arabidopsis [33]. One possible explanation for the discrepancy with our results based on two independent mutant alleles is that the constitutive pathogen-response phenotype is known to disappear under conditions of high humidity [57].

**Fig 6:**
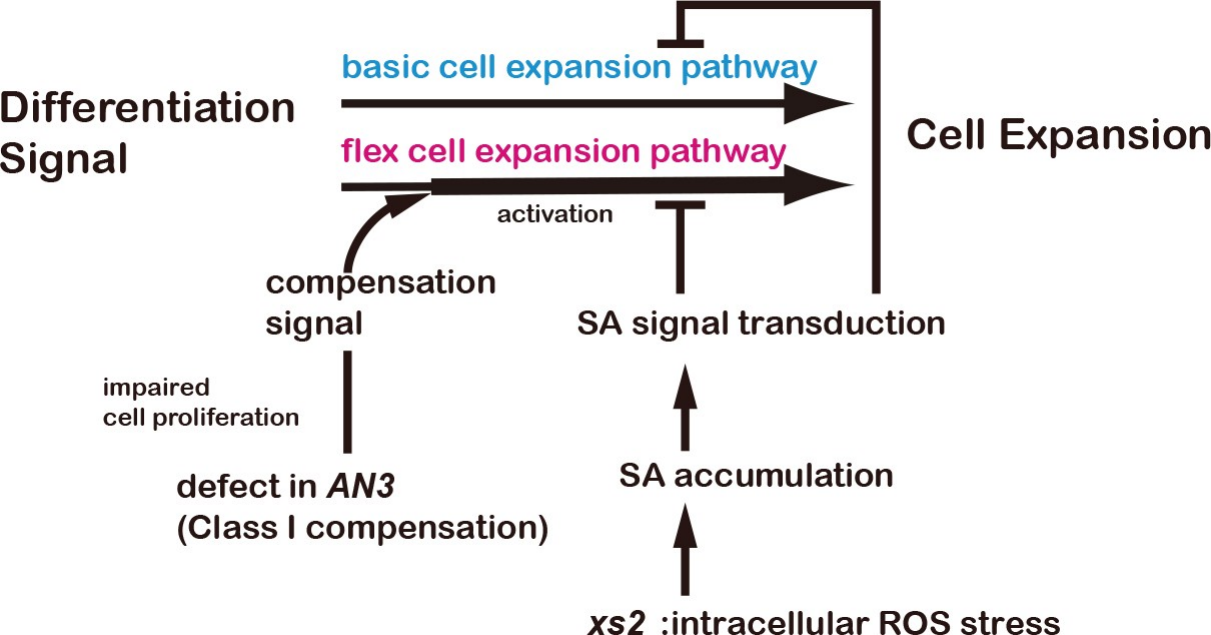
A schematic model of interaction between compensation and pathogen defense response on cell expansion.

A critical cue for the induction of compensation is a decrease in cell number below a certain threshold level [25]. Although our previous study [26] identified *xs* mutants that could suppress CCE in *an3* mutants, detailed molecular basis behind their suppression of CCE has been unknown. Our results show that CCE in class I compensation mutants is suppressed by introducing the *xs2* mutation, although the primary defects that trigger compensation differ between these mutants [23, 20]. Of note, activated SA signaling can suppress CCE in class I compensation mutants, but not in *KRP2ox*, a case of class III compensation, suggesting that the CCE in class I and class III are regulated by different regulatory networks. This result is consistent with previous reports that CCE in class I and III compensation are mediated by different pathways [13, 18, 19, 21]. Our results indicate that CCE in class I compensation occurs via the hyper-activation of a common cell expansion pathway that is also regulated negatively by SA signaling via an NPR1-dependent manner (Fig 5). It should be mentioned that CCE in *an3* was partially observed in the presence of 1 mM SA, rising the possibility that SA signaling and CCE regulate independent processes and SA-dependent suppression of CCE is additive phenotype. However, CCE in *xs2 an3* double mutant is completely suppressed and smaller cell phenotype in *xs2* is disappeared by introducing *npr1* mutation indicating CCE and SA signaling are involved in the same cell expansion pathway. Considering these results, we prefer to interpret that suppression of cell enlargement by SA in *an3* is not saturated or SA signaling partially suppress CCE pathway since level of CCE in the presence of SA is much moderate than control (Fig 4). Of course, at present we cannot discard the alternative idea that CCE in the *an3* and the SA signaling are independent, because SA is not downregulated (but slightly upregulated) in *an3* mutant. Further analyses will determine which is correct. Although the details of this cell expansion pathway are still unclear, our results highlight new insight of regulatory crosstalk between the basic plant development network and defense response (Fig 6).

## Materials and methods

### Plant materials and growth conditions

Arabidopsis accession Col-0 was used as WT for this study. *dnd1-1*, *npr1-1*, *ccx4-1* (SALK_113447) and *ccx4-2* (SALK_040272) were obtained from the Nottingham Arabidopsis Stock Centre (NASC; http://arabidopsis.info/). The *an3-2*, *an3-3*, *er-102*, *fugu2-1* and *KRP2ox* mutants have been described previously [13, 58]. The *xs2* mutant was originally isolated from the Col-0 background as described [11, 26]. T-DNA insertions and genotypes were confirmed by PCR amplification by using specific primers as described in the SIGnAL database (http://signal.salk.edu, S1 Table). The plants were grown under 16-h-light/8-h-dark conditions with white fluorescent illumination (approximately 48 μmol m^−2^ sec^−1^) at 22°C.

### Plant phenotyping

Leaf and cell sizes were measured as described [11, 26]. Values are represented as mean + SD. Each value corresponds to more than eight leaves sampled from 8 plants. See figure legends for further details of sample sizes. Student’s t-test or ANOVA followed by Tukey's HSD post-hoc test was performed to assess significant differences between the samples using the statistical software R (https://www.r-project.org/).

### SA treatment assay

To estimate the effect of SA on CCE suppression, SA was sprayed on 7-day-old seedlings with various concentrations (0 μM, 100 μM, 500 μM and 1 mM) in WT, *fugu2*, *an3*, *xs2*, *ccx4-1* and *KRP2ox* plants. SA spray was carried out continuously every day until leaves become mature (20-days-old plants).

### Genetic mapping

To map the *xs2* mutation, *xs2* in Col-0 background was crossed to Landsberg *erecta* and the resulting F_2_ population was used for mapping using molecular markers available from The Arabidopsis Information Resource (TAIR; http://www.arabidopsis.org/)). For the fine-mapping, more than 2000 F_2_ individuals were used. For critical recombinants, progeny testing was performed to verify the genotype at the mutant locus by analyzing the segregation of the phenotype in the progeny.

### Genotyping of T-DNA lines

Homozygous *ccx4-1* allele was obtained by PCR screening using newly designed LBb_new primers since LBb1.3 primer showed non-specific amplification in WT (S1 Table, S1C and S2 Figs). Since *ccx4-2* allele indicated multiple T-DNA insertions and could not separate them, we decided to use *ccx4-1* for further genetic analyses (S2 Fig).

### Trypan blue staining

The protocol followed Fernández-Bautista et al. [59]. Briefly, leaves were harvested with tweezers and immersed in fresh trypan blue staining solution (10 mg/ml trypan blue dissolved in solution mixed equal volume of lactic acid (85% w/w), phenol (TE buffered, pH 7.5–8.0), glycerol, and distilled water). After one-hour staining, leaves were washed with 99% ethanol, replacing ethanol several times until leaves were bleached. After the bleaching, leaves were mounted with glycerol solution (60% v/v) for the microscopy observation.

### Quantification of phytohormones

Phytohormones quantification was performed following the procedures described in Yoshimoto et al. [52].

### Expression analyses

To examine the expression of defense-response related genes, total RNA was isolated from leaves using the RNeasy plant mini kit (Qiagen). The isolated total RNA was treated with DNaseI (Takara) prior to the synthesis of first-strand cDNA by the SuperScript III first-strand synthesis system with oligo(dT)17 primer (Thermo). Primers used for expression analysis are listed in S1 Table. For internal control, *TUBULIN BETA 4* (*TUB4*) was used. Quantitative real-time RT-PCR analysis was performed using the THUNDERBIRD qPCR Mix (TOYOBO) with an Mx3000P QPCR System (Agilent Technologies). Average values from three technical and three biological replicates were shown.

## Supporting information

S1 Fig *ccx4-1* T-DNA mutant. (A) Position of the T-DNA insertion and primers which were used for genotyping. (B) Semi-quantitative RT-PCR analysis. Accumulation of *CCX4* and *CCX3* transcripts in WT and *xs2* and *ccx4-1* mutants are shown. Two individual samples from each genotype are shown. (C) Genotyping analysis in *xs2 ccx4-1* F1 progeny. T-DNA specific amplification (LBb_new-RP) and genomic DNA spanning T-DNA (LP-RP) are shown. (D) Schematic of fine-mapping showing the *XS2* locus on chromosome 1 to a 29.3 kb region. Genetic markers used in this study are indicated below the bars.
(TIF)

S2 Fig Genotyping and sequencing analysis in the *ccx4-1* and *ccx4-2* alleles. (A) Position of T-DNA transgene annotated on the database. (B) New primer for T-DNA genotyping. (C) Non-specific amplifications in LBb1.3-RP primer set in WT and *xs2* mutant. (D) Genotyping analysis with new primer in *ccx4-1*. (E) Genotyping analysis with new LBb primer in *ccx4-2* mutant. (F, G) Sequence analysis encompassing T-DNA borders in *ccx4-2* mutant by using LBb_new-LP PCR product (F) and LBb_new-RP PCR product (G). (H) Suggested situation of *CCX4* locus and location of T-DNA transgene in *ccx4-2*.
(TIF)

S3 Fig Trypan blue staining and leaf senescence phenotype. (A) Trypan blue staining in WT and *xs2*, and *ccx4-1* mutant. Arrowheads represent densely stained parts. (B) Cell area and number in WT, *an3-2*, *ccx4-1* and *an3-2 ccx4-1* mutant. (C) Leaf senescence phenotype for 32-days-old plants. Inflorescence stems were cut. Scale bars are 1 mm (A) and 10 mm (C).
(TIF)

S4 Fig Characterization of *dnd1*. (A) Cell size in WT, *dnd1* and *xs2*. (B) Images of mesophyll palisade cells from paradermal view. First leaves from three-weeks-old plants were used for observation. Means + SD. Scale bar is 50 μm.
(TIF)

S1 Table Primers used in this study. (TIF)
