# Correction: Suppression of class I compensated cell enlargement by xs2 mutation is mediated by salicylic acid signaling

**DOI:** 10.1371/journal.pgen.1010775

**Published:** 2023-05-19

**Authors:** Ushio Fujikura, Kazune Ezaki, Gorou Horiguchi, Mitsunori Seo, Yuri Kanno, Yuji Kamiya, Michael Lenhard, Hirokazu Tsukaya

In Fig 5, the authors showed xs2 npr1 double mutant phenotype to suggest that the suppression of cell expansion in the xs2 mutant was mediated via the NPR1-dependent pathway downstream of salicylic acid signaling. Incorrect lines were mistakenly used, resulting in the loss of the xs2 mutation. The authors have re-examined the correct double mutants of xs2 npr1 and found that the cell size in these double mutants was similar to that in xs2 single mutants. Please see the updated Fig 5 below.

**Fig 5:**
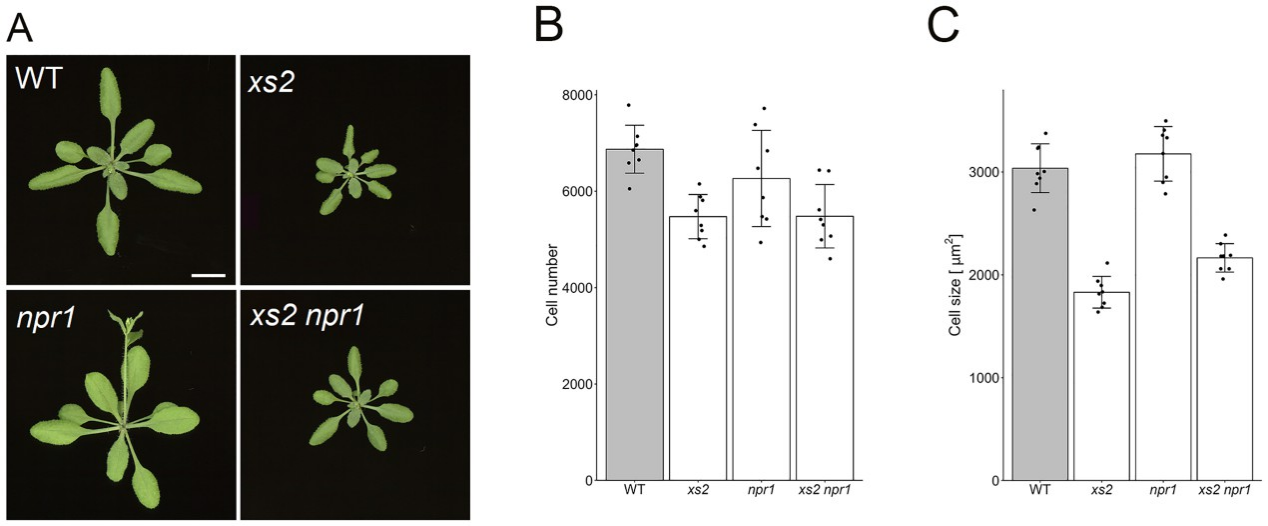
Characterization of the xs2 npr1 double mutant. (A) Rosette phenotype of xs2, npr1 and xs2 npr1 mutants. Plants were grown for three weeks under a 16-h-light/8-h-dark fluorescent illumination cycle at 22°C. Bars: 10 mm. (B) Estimated cell number and (C) cell size in WT and xs2, npr1 and xs2 npr1 mutants. First leaves from three-week-old plants were used for observation. (n ≥ 240 cells from more than eight leaves). Means + SD.
