# Supplementary figures and images for: Suppression of class I compensated cell enlargement by xs2 mutation is mediated by salicylic acid signaling
Source: PLoS Genet. 2020 Jun 25;16(6):e1008873. doi: 10.1371/journal.pgen.1008873 (PMC7343186; doi:10.1371/journal.pgen.1008873)

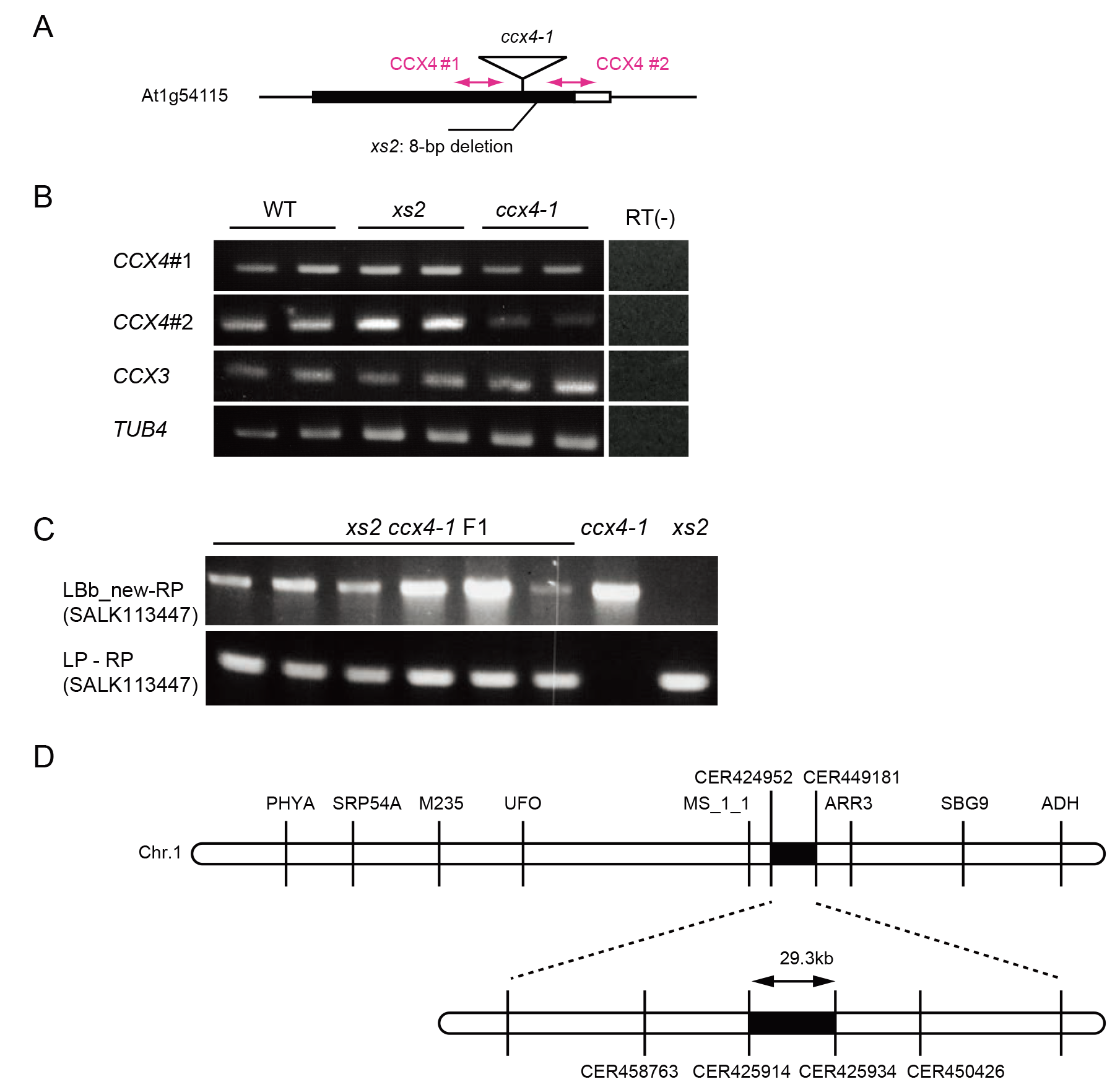

Supplement: S1 Fig — (A) Position of the T-DNA insertion and primers which were used for genotyping. (B) Semi-quantitative RT-PCR analysis. Accumulation of CCX4 and CCX3 transcripts in WT and xs2 and ccx4-1 mutants are shown. Two individual samples from each genotype are shown. (C) Genotyping analysis in xs2 ccx4-1 F1 progeny. T-DNA specific amplification (LBb_new-RP) and genomic DNA spanning T-DNA (LP-RP) are shown. (D) Schematic of fine-mapping showing the XS2 locus on chromosome 1 to a 29.3 kb region. Genetic markers used in this study are indicated below the bars. (TIF) [file pgen.1008873.s001.tif]

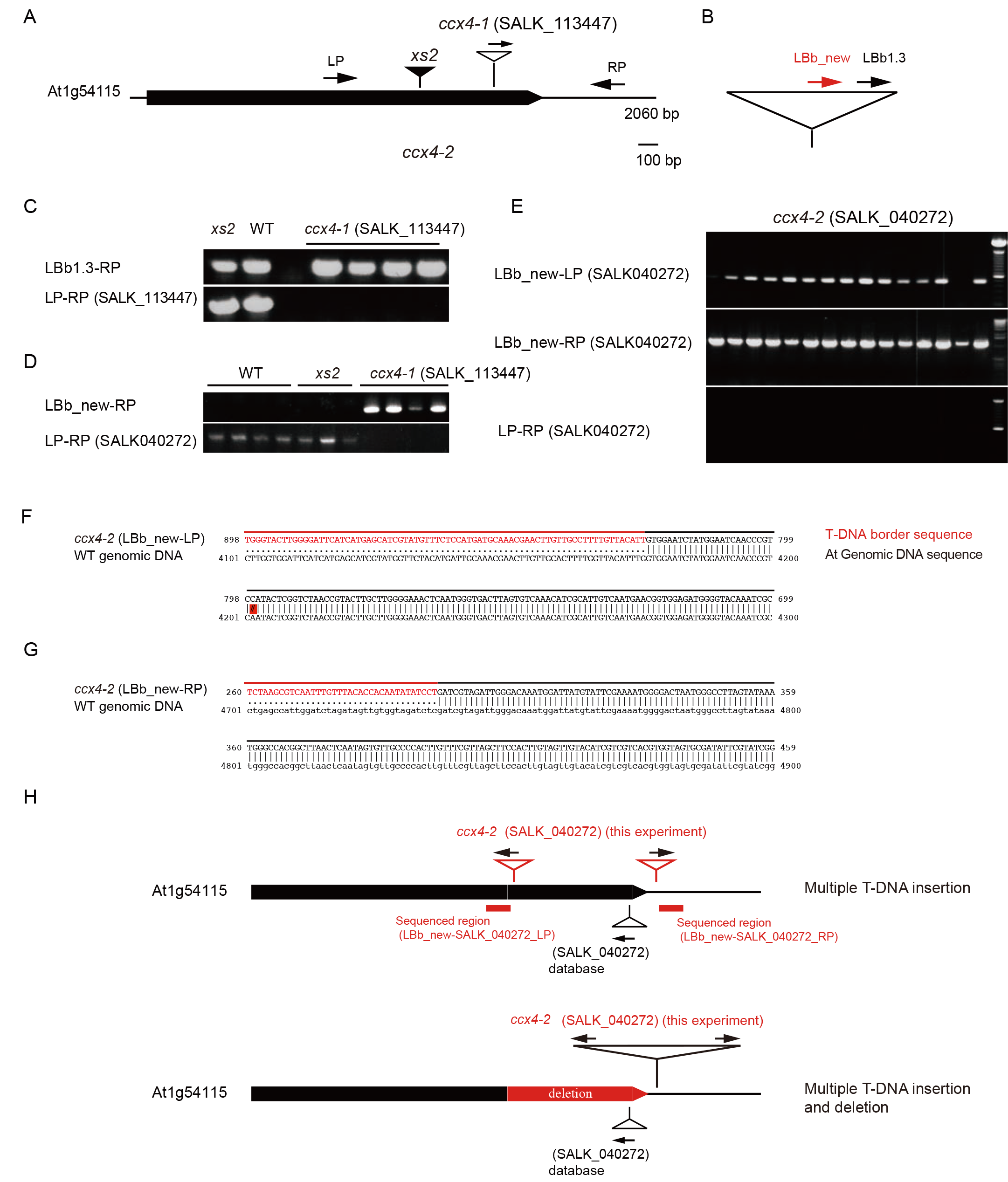

Supplement: S2 Fig — (A) Position of T-DNA transgene annotated on the database. (B) New primer for T-DNA genotyping. (C) Non-specific amplifications in LBb1.3-RP primer set in WT and xs2 mutant. (D) Genotyping analysis with new primer in ccx4-1. (E) Genotyping analysis with new LBb primer in ccx4-2 mutant. (F, G) Sequence analysis encompassing T-DNA borders in ccx4-2 mutant by using LBb_new-LP PCR product (F) and LBb_new-RP PCR product (G). (H) Suggested situation of CCX4 locus and location of T-DNA transgene in ccx4-2. (TIF) [file pgen.1008873.s002.tif]

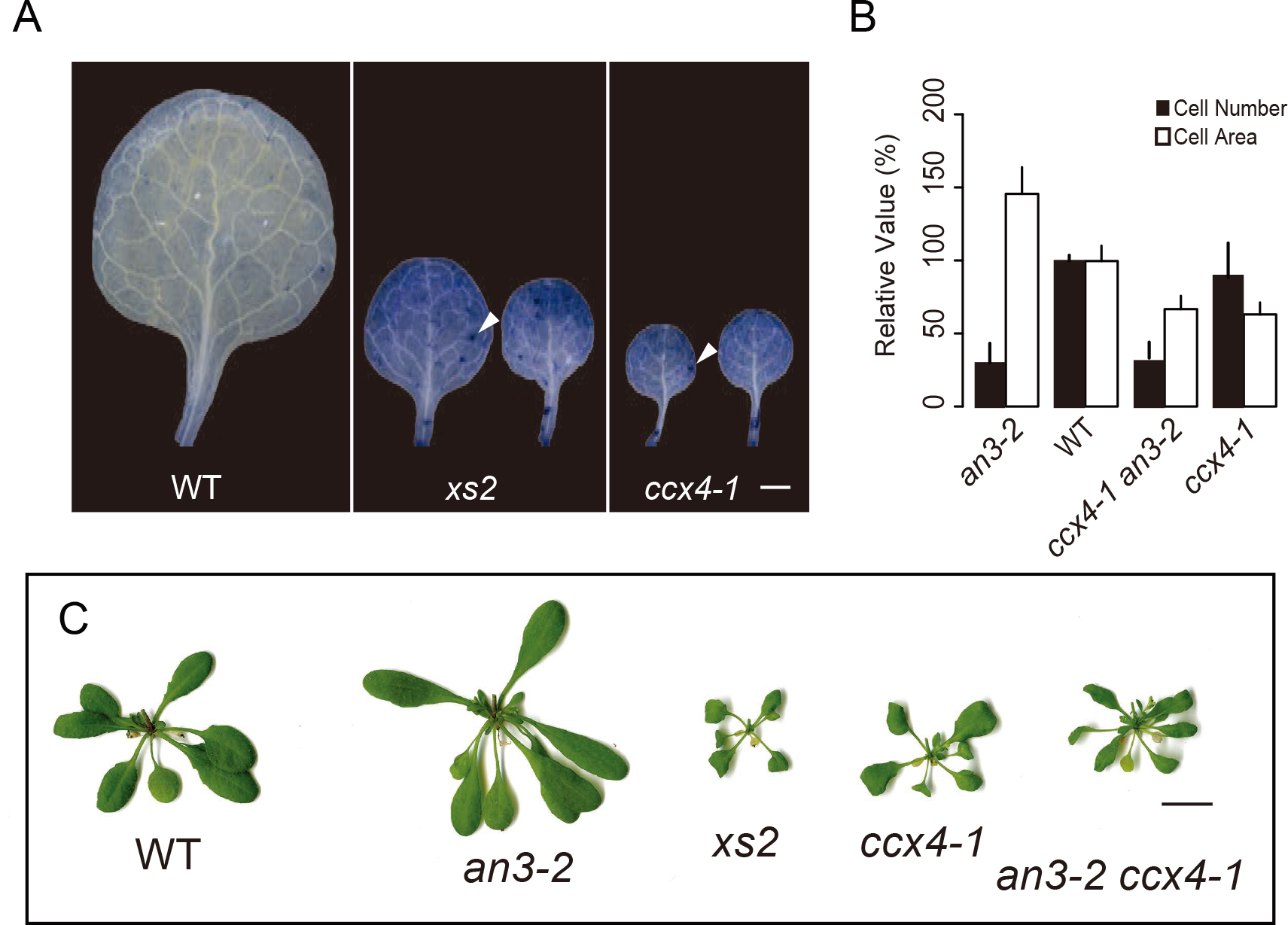

Supplement: S3 Fig — (A) Trypan blue staining in WT and xs2, and ccx4-1 mutant. Arrowheads represent densely stained parts. (B) Cell area and number in WT, an3-2, ccx4-1 and an3-2 ccx4-1 mutant. (C) Leaf senescence phenotype for 32-days-old plants. Inflorescence stems were cut. Scale bars are 1 mm (A) and 10 mm (C). (TIF) [file pgen.1008873.s003.tif]

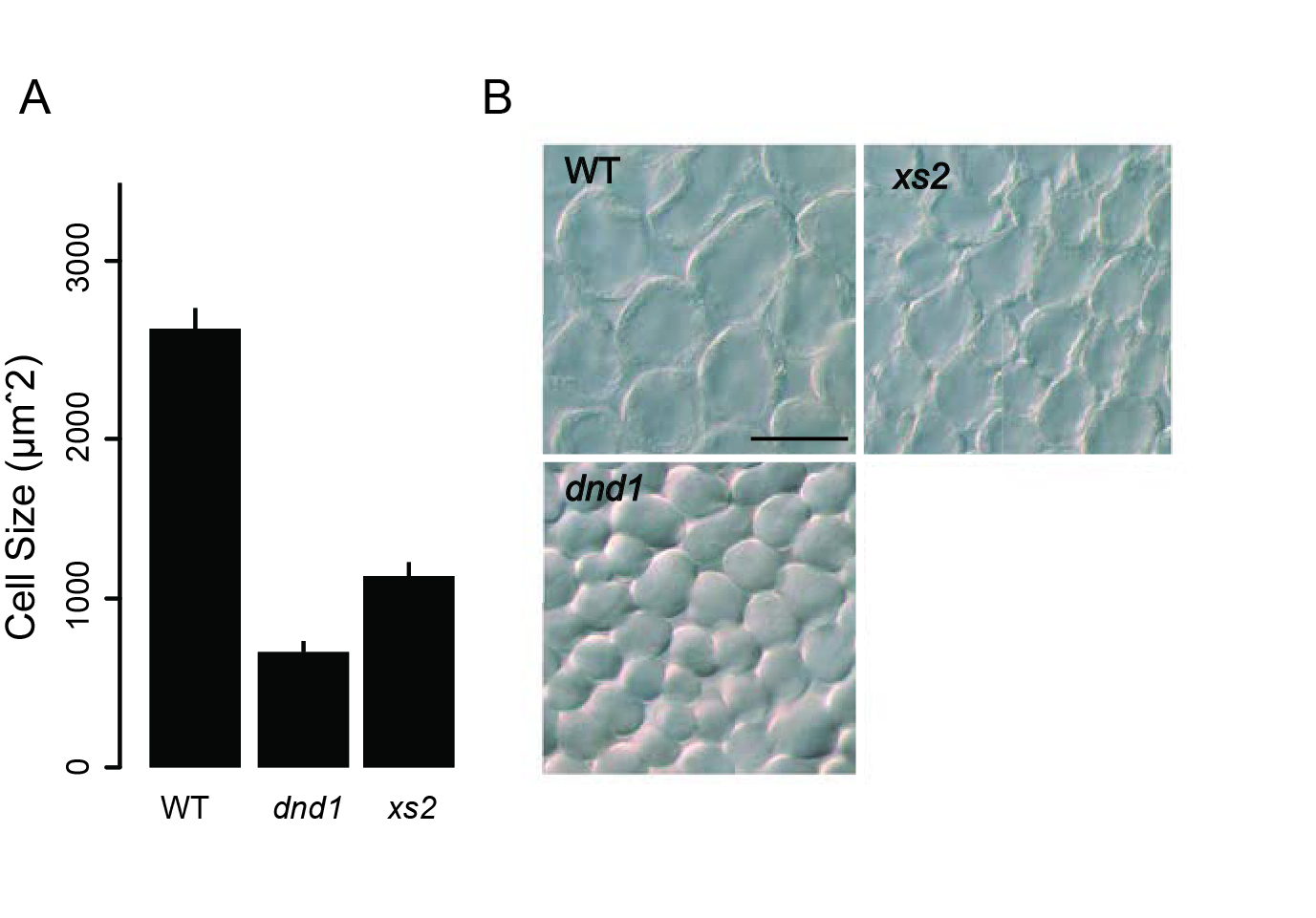

Supplement: S4 Fig — (A) Cell size in WT, dnd1 and xs2. (B) Images of mesophyll palisade cells from paradermal view. First leaves from three-weeks-old plants were used for observation. Means + SD. Scale bar is 50 μm. (TIF) [file pgen.1008873.s004.tif]

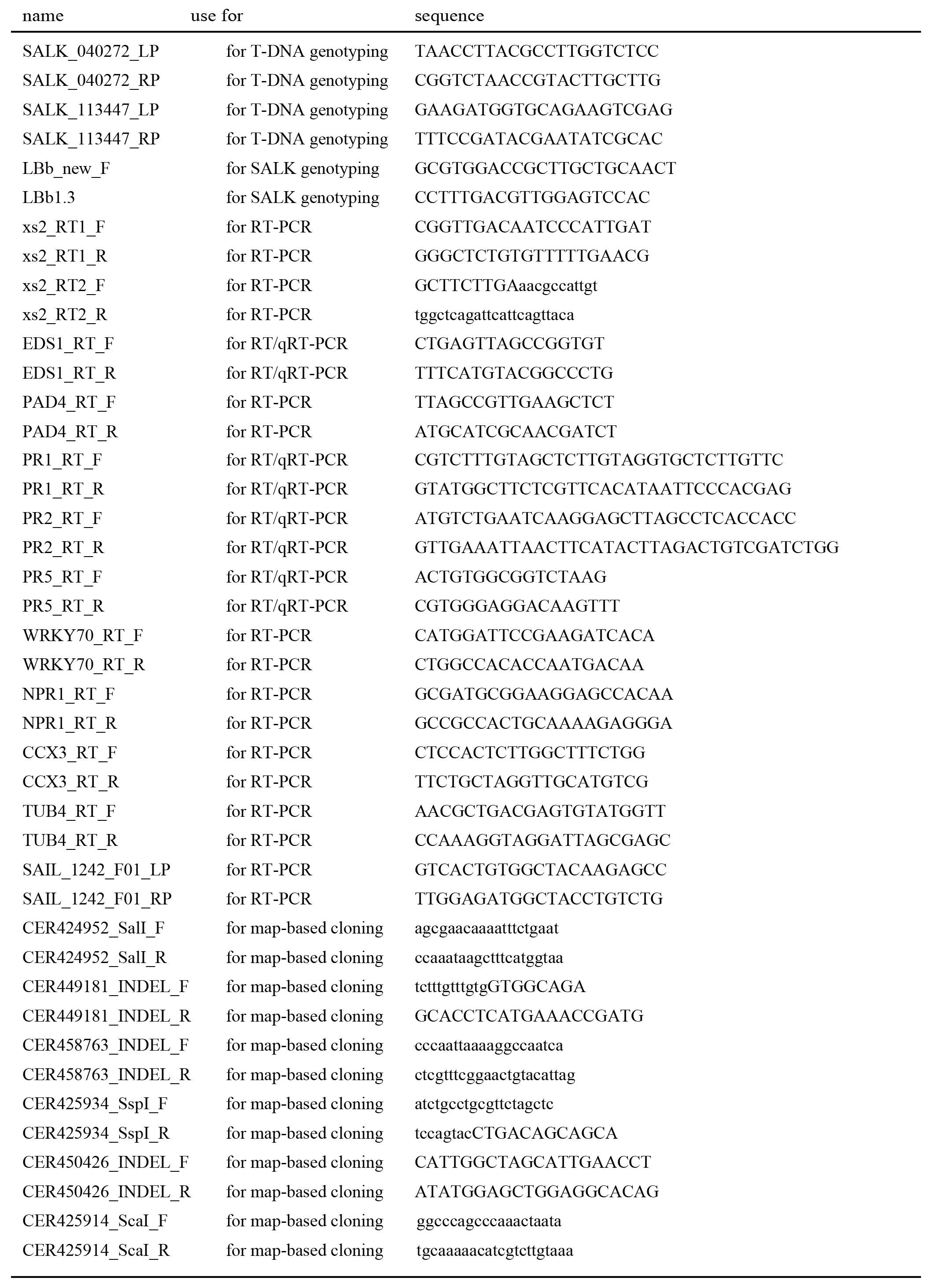

Supplement: S1 Table — (TIF) [file pgen.1008873.s005.tif]
